# Phyletic Distribution of Fatty Acid-Binding Protein Genes

**DOI:** 10.1371/journal.pone.0077636

**Published:** 2013-10-14

**Authors:** Yadong Zheng, David Blair, Janette E. Bradley

**Affiliations:** 1 School of Biology, University of Nottingham, Nottingham, United Kingdom; 2 State Key Laboratory of Veterinary Etiological Biology, Key Laboratory of Veterinary Parasitology of Gansu Province, Lanzhou Veterinary Research Institute, CAAS, Lanzhou, Gansu, China; 3 James Cook University, Townsville, Queensland, Australia; University of Lausanne, Switzerland

## Abstract

Fatty acid-binding proteins (FABPs) are a family of fatty acid-binding small proteins essential for lipid trafficking, energy storage and gene regulation. Although they have 20 to 70% amino acid sequence identity, these proteins share a conserved tertiary structure comprised of ten beta sheets and two alpha helixes. Availability of the complete genomes of 34 invertebrates, together with transcriptomes and ESTs, allowed us to systematically investigate the gene structure and alternative splicing of *FABP* genes over a wide range of phyla. Only in genomes of two cnidarian species could *FABP* genes not be identified. The genomic loci for *FABP* genes were diverse and their genomic structure varied. In particular, the intronless *FABP* genes, in most of which the key residues involved in fatty acid binding varied, were common in five phyla. Interestingly, several species including one trematode, one nematode and four arthropods generated FABP mRNA variants via alternative splicing. These results demonstrate that both gene duplication and post-transcriptional modifications are used to generate diverse FABPs in species studied.

## Introduction

Lipids are a very important subclass of constituents in the maintenance of normal physiology in organisms and a delicate balance of these hydrophobic molecules is partially regulated by fatty acid-binding proteins (FABPs). These small proteins of approximately 15 kDa execute fatty acid transport and, together with intracellular retinol- and retinoic acid-binding proteins, comprise a subfamily of intracellular lipid binding proteins (iLBPs) that are extensively present in animals. Ancestral iLBP genes are supposed to have arisen after separation of animals from fungi and plants [1]. FABPs are absent from archaebacteria and yeast [2,3]. Multiple gene duplications have occurred in this subfamily, giving rise to 16 iLBPs including 12 *FABP*s in vertebrates [1,4–6]. More than 30 *FABP* genes have been found in a wide range of invertebrates [2,7,8]. 

Mammalian *FABP* genes generally consist of four exons and some are dispersed on a single chromosome in humans, rats and mice [1,9]. The few studies on invertebrates show considerable variation in genomic organization of *FABP* genes, in aspects of size, exon and intron numbers [1,2]. For example, *Caenorhabditis elegans* expresses nine FABPs, also known as lipid binding proteins (LBP), and these mostly reside on different chromosomes. However, *LBP-5* and *LBP-6* are comprised of two exons and one intron and are positioned on chromosome I, suggesting that they might have arisen from tandem gene duplication.

Although FABPs share 20 to 70% identity at the amino acid level across and within invertebrate species, their tertiary structures are highly conserved, characterized by a cavity, formed by ten anti-parallel sheets and two helixes, that accommodates lipophilic compound(s), including fatty acids [2]. With a few exceptions, the residues related to ligand binding appear to be conserved in both invertebrate and vertebrate FABPs [9]. In the β-barrel cavity, the bound fatty acid(s) interacts with some residues Arg…Arg-x-Tyr, the so-called P2 motif. Moreover, Phe residues on the first helix and Ala/Pro-Asp in the turn between βE and βF are also critical for binding affinity in FABPs [10].

The systematic and genome-wide investigation of invertebrate *FABP* genes remains in its infancy. With availability of the complete genomes and transcriptome data for an increasing number of species, it is feasible to explore their genomic organization and post-transcriptional splicing paradigms. We have investigated gene organization and post-transcriptional modification of *FABP*s across 34 invertebrate species from 8 phyla (including lower chordates). Additionally, we have shown that an increase in gene copy numbers followed by divergence, as well as alternative splicing, are likely to be the mechanisms responsible for functional expansion and diversity of FABPs in invertebrate species. 

## Materials and Methods

### Identification and annotation of *FABP* genes

In this study, most of 34 invertebrates have annotated genomes and *FABP* genes were directly retrieved from the databases. For the species without an annotated genome including *Echinococcus multilocularis*, *Echinococcus granulosus*, *Heterorhabditis bacteriophora*，*Trichinella spiralis*，*Strongyloides ratti*，*Rhodnius prolixus*, *Haemonchus contortus* and *Ciona savignyi*, we searched the databases using the following strategies. Candidate *FABP* genes were identified using TBlastN, with experimentally or putatively identified *FABP* gene(s) from a closely related species as a query sequence, to search various genome databases with a cut-off e-value of 1-e10 (Table 1). Otherwise, *Schistosoma mansoni FABP*s (Smp_095360 and Smp_046800) or *C. elegans FABP*s (NP_505016, NP_508558, NP_508557, NP_491928, NP_506440, NP_491926, NP_001041249, NP_506444 and NP_001033511) were used as queries. This strategy was used because *FABP* genes share 20 to 70% similarity at the amino acid level. We then applied two criteria to resulting “hits” to identify *FABP* genes. First, considering that most known FABPs are ~130 amino acids (aa) in length, we arbitrarily set the size range of FABPs from 80 to 180aa (130 ± 50aa). In addition, the sequences within the size limit were used for secondary structure prediction and those with the putatively typical structural elements were considered to be *FABP* genes. 

**Table 1:** Distribution and features of *FABP* genes in invertebrates.

Species for which genome databases were searched	Num. loci found in genome drafts	Length^a^	Evidence^b^	Alternative splicing	Data origin^c^
**Cnidaria**					
*Nematostella vectensis*	/	/	/	/	JGI
*Hydra magnipapillata*	/	/	/	/	Metazome
**Placozoa**					
*Trichoplax adhaerens*	5	120~178	1/5	No	JGI NCBI
**Annelida**					
*Capitella teleta*	7	135~167	7/7	No	JGI NCBI
*Helobdella robusta*	3	119~143	3/3	No	JGI NCBI
**Mollusca**					
*Lottia gigantea*	7	132~163	7/7	No	JGI NCBI
**Platyhelminthes**					
*Schmidtea mediterranea*	3	123~168	2/3	No	SmedGD NCBI
*Schistosoma mansoni*	2	132, 133	2/2	Yes	GeneDB NCBI
*Schistosoma japonicum*	1	130	1/1	No	GeneDB NCBI
*Echinococcus granulosus*	5	124~143	2/5	No	NCBI Sanger
*Echinococcus multilocularis*	5	124~143	4/4	No	Sanger
**Nematoda**					
*Caenorhabditis elegans*	9	135~165	9/9	Yes	NCBI
*Pristionchus pacificus*	4	118~163	4/4	No	NCBI WormBase WUGSC
*Heterorhabditis bacteriophora*	3	133~164	3/3	No	NCBI WUGSC
*Trichinella spiralis*	3	133~143	3/3	No	NCBI WUGSC
*Haemonchus contortus*	0^d^	133~164	4/4	No	Sanger NCBI
*Strongyloides ratti*	4	132~165	4/4	No	Sanger WormBase
*Brugia malayi*	3	130~180	3/3	No	NCBI
**Arthropod**					
*Daphnia pulex*	2	130, 131	2/2	No	wFleaBase NCBI
*Pediculus humanus corporis*	3	132~135	0/3	No	NCBI VectorBase VectorBaseFlyBase
*Bombyx mori*	5	95~142	4/5	No	SilkDB
*Tribolium castaneum*	1	136	1/1	Yes	NCBI
*Nasonia vitripennis*	2	132	2/2	No	NCBI
*Acyrthosiphon pisum*	3	135, 136	3/3	Yes	NCBI
*Apis mellifera*	2	132, 133	2/2	Yes	NCBI
*Drosophila melanogaster*	1	130	1/1	Yes	NCBI FlyBase
*Anopheles gambiae*	2	131	1/1	No	VectorBase NCBI
*Aedes aegypti*	1	132	1/1	No	NCBI
*Culex pipiens quinquefasciatus*	1	132	1/1	No	NCBI
*Rhodnius prolixus*	1	134	1/1	No	NCBI VectorBase
**Echinodermata**					
*Strongylocentrotus purpuratus*	2	130	2/2	No	NCBI JGI
**Chordata**					
*Branchiostoma floridae*	15	135~151	7/14	No	JGI NCBI
*Ciona savignyi*	3	127~133	3/3	No	Broad NCBI
*Saccoglossus kowalevskii*	3	132~138	3/3	No	Baylor NCBI Metazome

a Number of amino acid residues;

b The number of putative *FABP* transcript variants (the numbers after ‘/’) and the number of the variants for which expression was validated by transcriptomic or/and EST data or/and cDNA cloning (the numbers before ‘/’);

c JGI: Joint Genome Institute; NCBI: National Centre for Biotechnology Information; SmedGD: *Schmidtea mediterranea* Genome Database; Sanger: Wellcome Trust Sanger Institute; WUGSC: Washington University Genome Sequencing Centre; wFleaBase: *Daphnia* Water Flea Genome Database; FlyBase: Drosophila database; SilkDB: silkworm database; Broad: Broad Institute; Baylor: Baylor College of Medicine;

d No genomic loci for *FBAP*s were found using Blast with its EST sequences.

Two sequential approaches were utilized for determination of the exons and exon boundaries. Firstly the exons and their boundaries were determined from TBlastN outcomes as highly-scored segment pairs or gaps within the segment pairs as described previously [11]. *FABP* gene structural models were then verified and finely modified using transcriptome data or expression sequence tags (ESTs). Segment pairs that dispersed over two or more supercontigs were not considered to build gene models in this study. The intron-exon boundaries were manually checked based on consensus splicing acceptor and donor sites and they conformed to the GT/AG rule. 

*FABP* genes, identified using the approach above, were used as query sequences to search transcriptome and EST databases for the relevant species. This provided a means of validating the findings from genomic data alone (Table 1). 

### Sequence alignments and secondary structure prediction

The FABP protein sequences were aligned using Clustal W algorithm (MEGA 4.0) with default parameters [12] and then manually checked (Figure S1). The secondary structures of FABPs were predicted using Psipred [13].

### Construction of a phylogenetic tree

Besides all the FABP amino acid sequences identified in this study, ten human FABP sequences were also included for phylogenetic analysis. Prior to tree construction, a best model was selected using TOPALi v2.5 [14]. A Bayesian tree was built using the following settings: WAG model plus gamma, 2 runs, 500,000 generations, 10 of sample frequency and 25% burn in. To confirm the topology of the tree, a ML tree was also built using the following settings: LG model plus gamma with 100 bootstraps.

## Results

### Identification and annotation of *FABP* genes across invertebrates

During sequence searching we obtained high-scoring hits that encoded more than 180aa or fewer than 80aa, but all of which were excluded from further analyses in this study. For instance, a *Branchiostoma floridae* hypothetical protein (987aa, XP_002589099) contained a region at the C terminal that shared 96% identity with *Branchiostoma belcheri* FABP (136aa, ADD10136). 

In total, 107 sequences falling within the specified size range and exhibiting appropriate secondary structure were collected from 32 invertebrate species including one placozoan, two annelids, one mollusc, five platyhelminths, seven nematodes, twelve arthropods, one echinoderm and three chordates (Table 1 and Supplementary text file). The identity of these putative FABP amino acid sequences ranged from 29.0% to 99.3% and they were predicted to have the typical tertiary structure (Figure S2). No homologues of *FABP*s were identified in two Cnidaria species, *Hydra magnipapillata* and *Nematostella vectensis*. Notably, four *Haemonchus contortus FABP* genes were identified by TBlastN searches against transcriptome (NCBI) but none of them was found in the genome, possibly due to incomplete genomic data (Sanger). One putative FABP transcript was derived from transcriptome or EST data, but its locus was not found in the genome of each of the following species: *Helobdella robusta, Lottia gigantea, Schistosoma japonicum, Heterorhabditis bacteriophora* and *Saccoglossus kowalevskii*. With the exception of the body louse, *Pediculus humanus corporis*, some or all *FABP* genes found in genomes were validated by EST or transcriptomic data.

 Numbers of genomic loci for *FABP*s ranged from one (several arthropods and *S. japonicum*) to fifteen (the chordate, *B. floridae*) in invertebrate genomes (Table 1). *Echinococcus multilocularis, Anopheles gambiae* and *B. floridae* each had two distinct loci that encoded identical FABPs at the amino acid level. The introns of the two *E. multilocularis FABP*s were identical, whilst those of the *A. gambiae* and *B. floridae* FABPs were different in size and sequence. But there is not enough evidence to support that these *FABP* genes are transcribed into the same mRNAs.

### Phylogenetic analysis of *FABP*s

**Figure 1:**
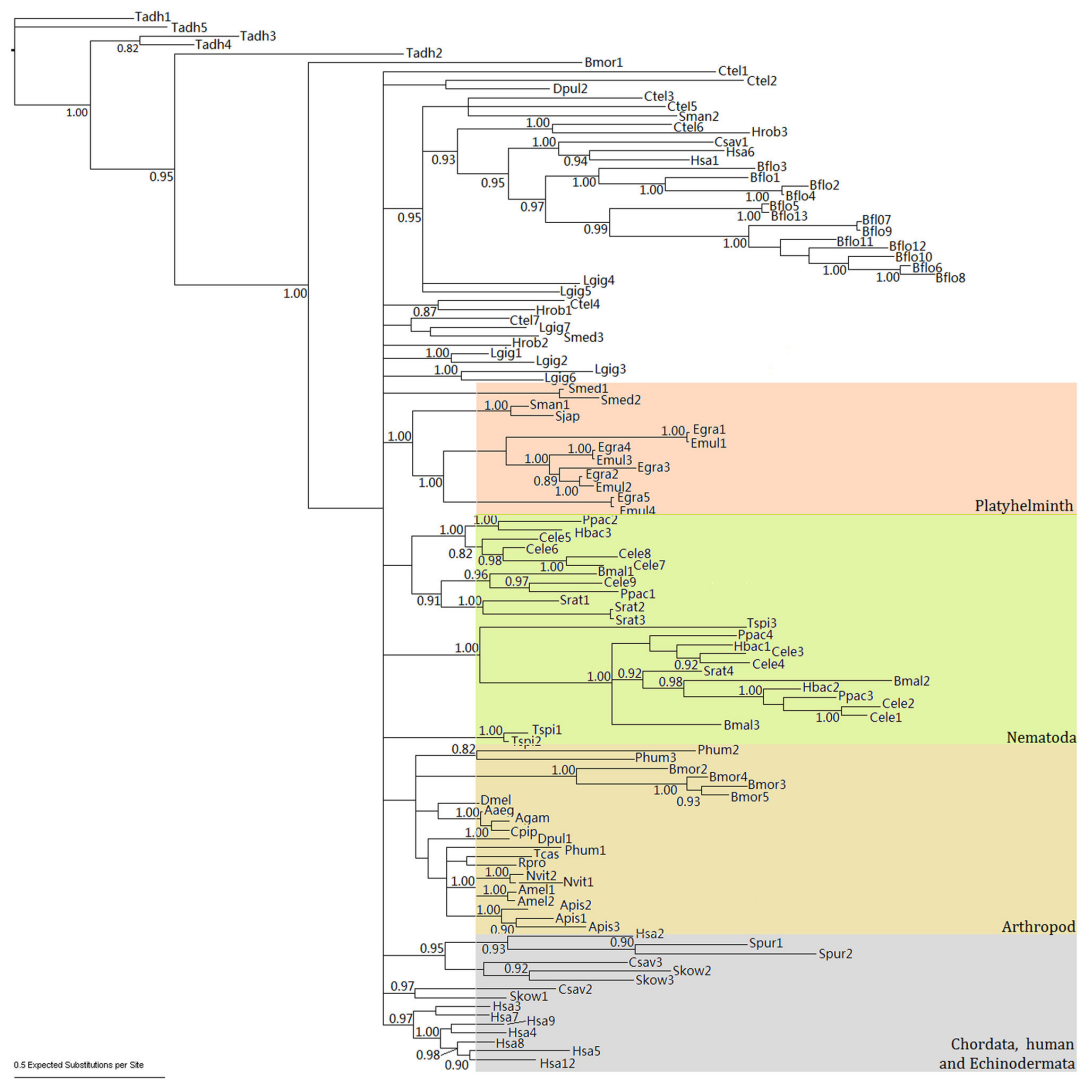
A Bayesian tree of FABPs. Bayesian probabilities more than 0.8 were shown at nodes. Tadh: *Trichoplax adhaerens*; Ctel: *Capitella* *teleta*; Hrob: *Helobdella robusta*; Lgig: *Lottia gigantean*; Smed: *Schmidtea mediterranea*; Sman: *Schistosoma mansoni*; Sjap: *Schistosoma japonicum*; Egra: *Echinococcus granulosus*; Emul: *Echinococcus multilocularis*; Cele: *Caenorhabditis elegans*; Ppac: *Pristionchus pacificus*; Hbac: *Heterorhabditis bacteriophora*; Tspi: *Trichinella spiralis*; Srat: *Strongyloides ratti*; Bmal: *Brugia malayi*; Dpul: *Daphnia pulex*; Phum: *Pediculus humanus corporis*; Bmor: *Bombyx mori*; Tcas: *Tribolium castaneum*; Nvit: *Nasonia vitripennis*; Apis: *Acyrthosiphon pisum*; Amel: *Apis mellifera*; Dmel: *Drosophila melanogaster*; Agam: *Anopheles gambiae*; Aaeg: *Aedes aegypti*; Cpip: *Culex pipiens quinquefasciatus*; Rpro: *Rhodnius prolixus*; Spur: *Strongylocentrotus purpuratus*; Bflo: *Branchiostoma floridae*; Csav: *Ciona savignyi*; Skow: *Saccoglossus kowalevskii*; Has: *Homo sapiens* Note: to make it simpler, ‘FABP’ was omitted in every branch name. For example: Tadh1 refers to Tadh_FABP1, Tadh2 to Tadh_FABP2 and so forth.

As shown in the Bayesian tree (Figure 1), nematode FABPs formed two distant clades and with an exception of *T. spiralis*, each clade was comprised of all the nematode species, suggesting that the *FABP* genes in nematodes may have evolved from different origins. Except *S. mediterranea*, the phylogenetic relationship within Platyhelminth species was clearly resolved. The subclades comprised of *E. multilocularis* and *E. granulosus* demonstrate that both parasites have a similar gene set for *FABP*, possibly descendent from their common ancestor.

Extraordinary gene expansion was observed in amphioxus, *B. floridae*, via gene duplications. Moreover, the phylogenetic analysis revealed that the current gene set might have resulted from multiple rounds of duplications and divergence during evolution, especially Bflo11 paralogues, and that these duplication events might have occurred recently (Figure 1). Essentially, a ML tree showed a similar topology to the Bayesian tree (Figure S3).

### Diversity of *FABP* gene structures across invertebrates

Although intronless *FABP* pseudogenes have been described in several species including humans [15–17], all functional mammalian *FABP* genes exhibit similar genomic organization, containing four exons and three introns [1]. An analysis of *FABP* gene organization revealed diversity in invertebrates, especially in Platyhelminthes and Nematoda, although the canonical organization (four exons) predominated. A six-exon five-intron structure for FABP was only found in the early-branching invertebrate *Trichoplax adhaerens*. *FABP* genes comprised of five exons and four introns were found in placozoans, molluscs, platyhelminths and nematodes (Table 2). 

**Table 2:** FABP genomic structures in invertebrates.

Species^a^	Number of exons					
	6	5	4	3	2	1
**Placozoa**						
*Trichoplax adhaerens*	1	1	3			
**Annelida**						
*Capitella teleta*			7			
*Helobdella robusta*			2			
**Mollusca**						
*Lottia gigantea*		1	7			
**Platyhelminthes**						
*Schmidtea mediterranea*		1	2			
*Schistosoma mansoni*			2			
*Schistosoma japonicum*				1		
*Echinococcus granulosus*					3	2
*Echinococcus multilocularis*					3^b^	2
**Nematoda**						
*Caenorhabditis elegans*			1	4	4	
*Pristionchus pacificus*		1	2			
*Heterorhabditis bacteriophora*		2	1			
*Trichinella spiralis*			3			
*Strongyloides ratti*					1	3
*Brugia malayi*			2	1		
**Arthropod**						
*Daphnia pulex*			1	1		
*Pediculus humanus corporis*				1		2
*Bombyx mori*			4	1		
*Tribolium castaneum*				1		
*Nasonia vitripennis*				2		
*Acyrthosiphon pisum*				3		
*Apis mellifera*			1	1		
*Drosophila melanogaster*				1		
*Anopheles gambiae*					2^b^	
*Aedes aegypti*					1	
*Culex pipiens quinquefasciatus*					1	
*Rhodnius prolixus*						1
**Echinodermata**						
*Strongylocentrotus purpuratus*					1	1
**Chordata**						
*Branchiostoma floridae*			12^b^	3		
*Ciona savignyi*			2	1		
*Saccoglossus kowalevskii*			1			3
**Total**	1	6	52	22	16	14

a In total thirty-four species from eight phyla were included in this study;

b Each of these species has two different loci that encode identical FABPs at the amino acid level.

### Intronless *FABP* genes

Single-exon *FABP* genes were found in the following species: *Echinococcus granulosus*, *E. multilocularis*, *Strongyloides ratti*, *P. humanus corporis*, *Rhodnius prolixus*, *Strongylocentrotus purpuratus* and *S. kowalevskii* (Table 2). Expression of most of these intronless genes was confirmed either by transcriptome analysis or analysis of ESTs. With the exception of *R. prolixus*, the species encoding intronless *FABP*s also encoded other *FABP* genes with two or more exons. The intronless *FABP* gene architecture dominated in three other metazoans *Strongyloides ratti*, *P. humanus corporis* and *Saccoglossus kowalevskii*.

Alignment of the intronless FABPs has revealed that the *R. prolixus* FABP contained intact key residues which are important in defining fatty acid binding [10], while absence or alterations in these sites occurred in the others (Figure S4). This suggests that *R. prolixus* FABP has capacity to bind to lipids, but the others may no longer be able to do so. Alternatively, they may have different binding spectra for fatty acids in comparison with those that have been characterised. 

### Alternative splicing in *FABP* genes

Alternative splicing is an important post-transcriptional modification in eukaryotic pre-mRNAs, accounting for the complexity and variety of proteomes. *FABP* genes underwent alternative splicing to generate isoforms in several invertebrates including *S. mansoni*, *C. elegans*, *T. castaneum*, *Acyrthosiphon pisum*, *Apis mellifera* and *Drosophila melanogaster*. Furthermore, all the transcripts derived from alternative splicing were confirmed by transcriptomic data. 

**Figure 2:**
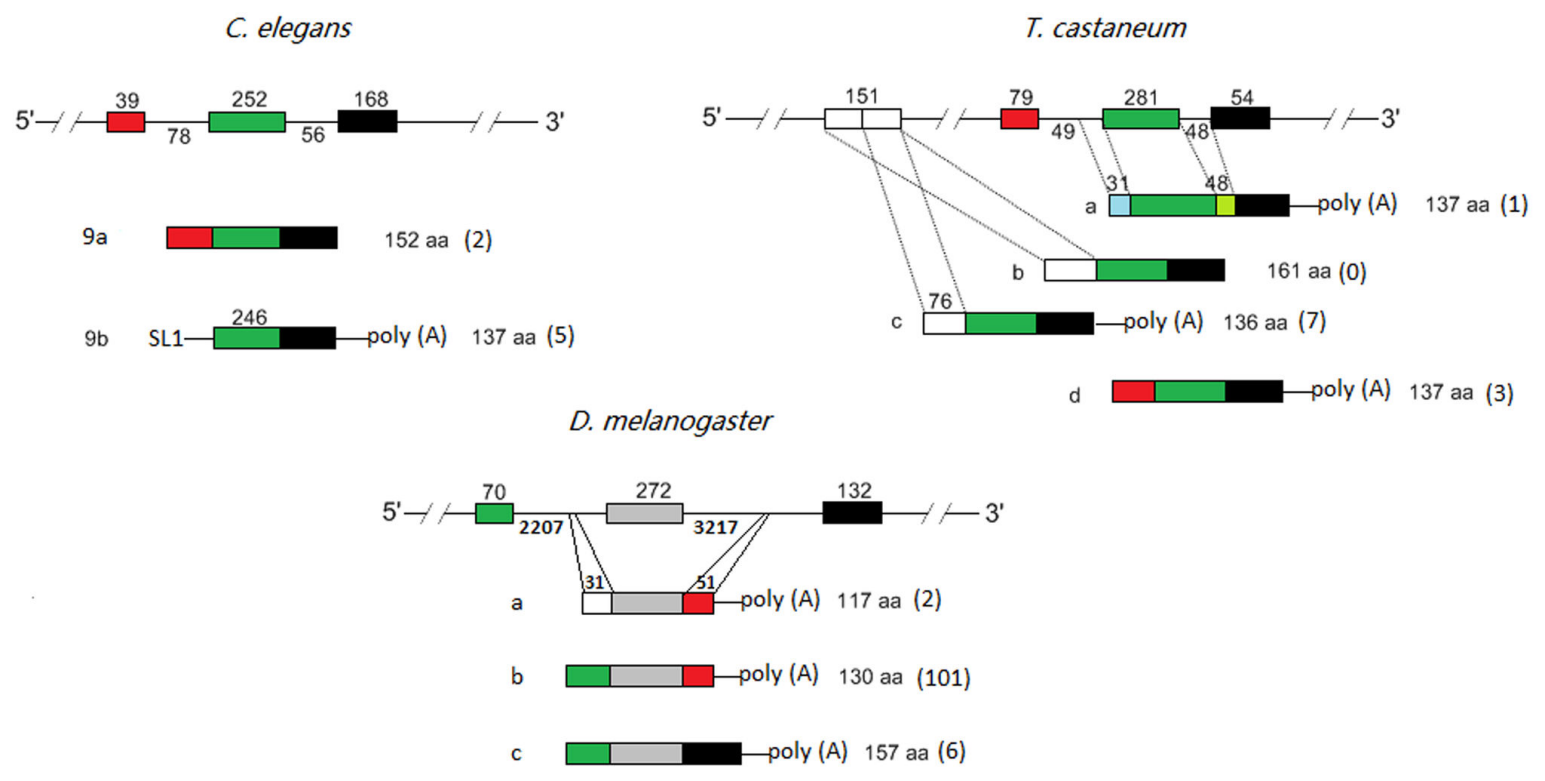
Alternative splicing in invertebrate *FABP* genes. Typical alternative splicing patterns in *C. elegans*, *T. castaneum* and *D. melanogaster* are represented. In *C. elegans*, LBP-9 pre-mRNA is spliced to generate two variants 9a and 9b by addition of a short spliced leader sequence (SL1: 5’-GGTTTAATTACCCAAGTTTGAG-3’) at the 5’ end. Blank or filled boxes and straight lines represent exons and introns, respectively, and a poly (A) stretch present in each *FABP* cDNA clone or EST sequence is directly shown. In each group, an annotated *FABP* gene is placed above the variants that are indicated by a, b, c or/and d. Numbers above the boxes and under the lines show the sizes of corresponding exons and introns, respectively. The sizes of exons, where these differ, are indicated above the corresponding exons in the spliced transcripts. The length of variants is also shown after each transcript and the number of ESTs is shown in the brackets.

Comparative analysis of *FABP* gene structure and transcripts revealed that *FABP* pre-mRNAs were alternatively spliced in different patterns (Figure 2). In *S. mansoni*, four *FABP* variants were produced via exon skipping. Moreover, these variants were differentially expressed at different developmental stages (http://www.genedb.org/Homepage/Smansoni), suggesting they have distinct roles. The arthropods *Acyrthosiphon pisum* (3 *FABP* genes) and *Apis mellifera* (2 *FABP* genes) utilized the same approach to yield four and three different *FABP* transcripts, respectively. In contrast to the exon exclusion mechanism seen in *S. mansoni*, spliced leader trans-splicing (SL *trans*-splicing), a type of alternative splicing whereby a spliced leader serves as a mini-exon to be added onto 5’ pre-mRNA ends [18], was used in *C. elegans FABP* mRNA precursors (Figure 2). 

Both *T. castaneum* and *D. melanogaster* genomes contained only one *FABP* gene locus but variants were found in transcriptome or EST datasets. Mapping of these transcripts revealed that another alternative splicing mechanism, intron retention, was involved in the post-transcriptional splicing of *FABP* genes together with exon skipping (Figure 2). Noticeably, the last exons of the *FABP* genes were retained during alternative splicing in all the invertebrates studied except *D. melanogaster* that also used the partial sequence of the second intron as the last exon to generate *FABP* isoforms.

## Discussion

In this study, the number of the *FABP* genomic loci identified was remarkably variable, from 1 in several invertebrates to 15 in *B. floridae*. Interestingly, no *FABP* loci were identified in genomes of the cnidarians *H. magnipapillata* and *N. vectensis*, yet they were present in the simplest known free-living metazoan, *T. adhaerens*, which is considered as a basal metazoan [19,20]. Consistent with the gene structure, *T. adhaerens FABP* genes seem to be prototypes of this family (most loci exhibit the “canonical” four-exon structure). The lack of FABP expression in cnidarians may be explained by gene loss but the possibility remains that these species may express extremely heterogeneous FABPs and investigations of more cnidarians are required. 

Also of interest is the finding that each of three invertebrate genomes contained two loci to encode the same proteins, suggesting that they might have arisen from recent gene duplication. A phylogenetic analysis suggested that the current gene set in *E. multilocularis* may have been generated before the speciation of *Echinococcus* species (Figure 1), supporting the idea that *FABP* gene duplication may have occurred in their common ancestor. This finding does not fully support the previous assumption that *E. granulosus FABP*s 2 and 4 arose from a recent duplication event [21].

The ancestral *FABP* gene might have evolved from a lipocalin gene and have undergone the first duplication approximately 930 million years ago with subsequent duplications and divergence [1,22]. The *FABP* sequences annotated in this study were heterogeneous with regard to length, composition and identity, possibly driven by the need to transport numerous different fatty acids [1]. In contrast to fifteen *FABP* copies in the lower chordate *B. floridae*, arthropods often possessed only a single *FABP* locus. This was the situation in two species of mosquito, *Aedes aegypti* and *C. pipiens quinquefasciatus*. However, a malaria mosquito, *Anopheles gambiae*, had two copies of *FABP* genes that resided on the same scaffold. It is noteworthy that mosquito *FABP*s have been annotated as allergens (XP_001657349 for *Aedes aegypti* FABP; XP_001864031 for *C. pipiens quinquefasciatus* FABP) [23]. Although no evidence is to date available, it is possible that mosquito FABPs act as allergens. FABPs from mites [24,25] and other lipid-binding proteins from nematodes [26–28] have been shown to be allergic. Secondary structural prediction with high confidence showed that all of these mosquito allergens had two alpha helixes and ten beta sheets typical of *FABP* structural elements (data not shown). In addition to the conserved secondary structures, they contained fatty acid binding-related key residues except Val-Asp instead of Pro-Asp (Figure S4). We therefore propose that these allergens in mosquitoes are functional FABPs. 

In contrast to relatively uniform genomic structures for mammalian *FABP* genes, invertebrate *FABP* genes were organized in a wide range of patterns with a dominance of the four-exon three-intron structure. This study indicates that invertebrate *FABP* genes may have tended towards loss of introns during evolution. This idea is enhanced by the fact that most of the invertebrate *FABP*s investigated have matched intron positions [2]. Compared to the early branching invertebrate, *T. adhaerens*, *FABP* genes from cestodes and mosquitoes were intron-poor. Our findings strongly argue against the speculation that the first and second introns of *FABP* genes might have evolved later [21]. Surprisingly, a number of invertebrates encoded intronless *FABP*s with most of the key residues that participate in lipid binding being altered. Here no evidence was obtained to suggest that these FABPs remain able to bind to lipids. However, with the exception of *E. granulosus* and *P. humanus corporis*, transcription of intronless *FABP*s in other species was verified by transcriptomic or/and EST data, suggesting that they are functional. Such intronless *FABP*s have also been reported in several mammals where they may have lost their capacity to bind lipid ligands although it has not been fully established if they are transcribed [15–17]. 

A wealth of data has revealed that numerous introns were present generally in early multicellular organisms and alterations of intron positions occurred at a very low frequency during evolution [29]. Several mechanisms have been proposed for intron gain or loss [30,31]. In comparison with a canonical three-intron structure, the first intron (17/31) was more likely to be preferentially retained in two- or one-intron *FABP* genes in invertebrates. This suggests that reverse transcription followed by gene conversion may have been involved in the *FABP* intron loss as this mechanism tends to remove 3’ introns from genes [30]. An analysis of 684 gene introns from eight organisms has showed that loss of most ancestral introns has occurred in worms and arthropods but not in humans [32]. This result may give us some clues, but the selective forces that have driven intron loss in platyhelminths remain unclear. 

Alternative splicing, a substantial mechanism for the modification of pre-mRNA, exists in nearly all eukaryotic organisms and accounts for the complexity and diversity of protein functions. In contrast to mammals, where alternative splicing of *FABP* genes has rarely been observed, *FABP* genes in some invertebrates were alternatively spliced, leading to generation of FABP variants. In particular, these various transcripts were produced by different splicing patterns. In *C. elegans*, only *FABP* genes 5, 6 and 9 were confirmed to mature by means of SL trans-splicing using spliced leader 1 (SL1). There are two distinct spliced leader sequences in *C. elegans*, SL1 and SL2, and the former is used to generate mainly monocistronic pre-mRNA [33]. It is estimated that approximately 70% of all genes in this free-living nematode are post-transcriptionally modified by this mechanism [34]. It is still not clear why *C. elegans FABP 1, 2,* 3 and 8 pre-mRNAs are not matured via SL trans-splicing. Although the SL trans-splicing mechanism is also extensively present in the Phyla Cnidaria, Platyhelminthes and Chordata [18], it was not observed in FABP transcripts in other invertebrates collected in this study. These results suggest that the SL trans-splicing modification in *FABP* transcripts may have been acquired during evolution of *C. elegans*.

## Supporting Information

Figure S1 **Alignment of FABP amino acid sequences.** Tadh: *Trichoplax adhaerens*; Ctel: *Capitella* *teleta*; Hrob: *Helobdella robusta*; Lgig: *Lottia gigantean*; Smed: *Schmidtea mediterranea*; Sman: *Schistosoma mansoni*; Sjap: *Schistosoma japonicum*; Egra: *Echinococcus granulosus*; Emul: *Echinococcus multilocularis*; Cele: *Caenorhabditis elegans*; Ppac: *Pristionchus pacificus*; Hbac: *Heterorhabditis bacteriophora*; Tspi: *Trichinella spiralis*; Srat: *Strongyloides ratti*; Bmal: *Brugia malayi*; Dpul: *Daphnia pulex*; Phum: *Pediculus humanus corporis*; Bmor: *Bombyx mori*; Tcas: *Tribolium castaneum*; Nvit: *Nasonia vitripennis*; Apis: *Acyrthosiphon pisum*; Amel: *Apis mellifera*; Dmel: *Drosophila melanogaster*; Agam: *Anopheles gambiae*; Aaeg: *Aedes aegypti*; Cpip: *Culex pipiens quinquefasciatus*; Rpro: *Rhodnius prolixus*; Spur: *Strongylocentrotus purpuratus*; Bflo: *Branchiostoma floridae*; Csav: *Ciona savignyi*; Skow: *Saccoglossus kowalevskii*; Has: *Homo sapiens* Note: to make it simpler, ‘FABP’ was omitted in every branch name. For example: Tadh1 refers to Tadh_FABP1, Tadh2 to Tadh_FABP2 and so forth.
(TIF)

Figure S2 **Tertiary structure of Emul_FABP3 predicted using Phyre.**
(TIF)

Figure S3 **A ML tree of FABPs.** Bootstrap values more than 60 were shown at nodes. Tadh: *Trichoplax adhaerens*; Ctel: *Capitella* *teleta*; Hrob: *Helobdella robusta*; Lgig: *Lottia gigantean*; Smed: *Schmidtea mediterranea*; Sman: *Schistosoma mansoni*; Sjap: *Schistosoma japonicum*; Egra: *Echinococcus granulosus*; Emul: *Echinococcus multilocularis*; Cele: *Caenorhabditis elegans*; Ppac: *Pristionchus pacificus*; Hbac: *Heterorhabditis bacteriophora*; Tspi: *Trichinella spiralis*; Srat: *Strongyloides ratti*; Bmal: *Brugia malayi*; Dpul: *Daphnia pulex*; Phum: *Pediculus humanus corporis*; Bmor: *Bombyx mori*; Tcas: *Tribolium castaneum*; Nvit: *Nasonia vitripennis*; Apis: *Acyrthosiphon pisum*; Amel: *Apis mellifera*; Dmel: *Drosophila melanogaster*; Agam: *Anopheles gambiae*; Aaeg: *Aedes aegypti*; Cpip: *Culex pipiens quinquefasciatus*; Rpro: *Rhodnius prolixus*; Spur: *Strongylocentrotus purpuratus*; Bflo: *Branchiostoma floridae*; Csav: *Ciona savignyi*; Skow: *Saccoglossus kowalevskii*; Has: *Homo sapiens* Note: to make it simpler, ‘FABP’ was omitted in every branch name. For example: Tadh1 refers to Tadh_FABP1, Tadh2 to Tadh_FABP2 and so forth.
(TIF)

Figure S4 **Fatty acid binding-related residues in intronless FABP genes of invertebrates.** Invertebrate intronless FABP amino acid sequences were aligned using Clustal W. The amino acids identical to the consensus are shown as dots and alignment gaps are indicated with dashes (-). Numbers above the alignment represent positions of amino acids. The key amino acids responsible for interactions with lipid ligands are directly indicated beneath the alignment. Rpro, *Rhodnius prolixus*; Egra, *Echinococcus granulosus*; Emul, *E. multilocularis*; Srat, *Strongyloides ratti*; Phum, *Pediculus humanus corporis*; Spur, *Strongylocentrotus purpuratus*; Skow, *Saccoglossus kowalevskii*. Note: to make it simpler, ‘FABP’ was omitted in every branch name. For example: Tadh1 refers to Tadh_FABP1, Tadh2 to Tadh_FABP2 and so forth.
(TIF)

File S1 **Supplementary text file**. Putative amino acid sequences of *FABP* genes.
(TXT)
