# Supplementary figures and images for: Phyletic Distribution of Fatty Acid-Binding Protein Genes
Source: PLoS One. 2013 Oct 14;8(10):e77636. doi: 10.1371/journal.pone.0077636 (PMC3796463; doi:10.1371/journal.pone.0077636)

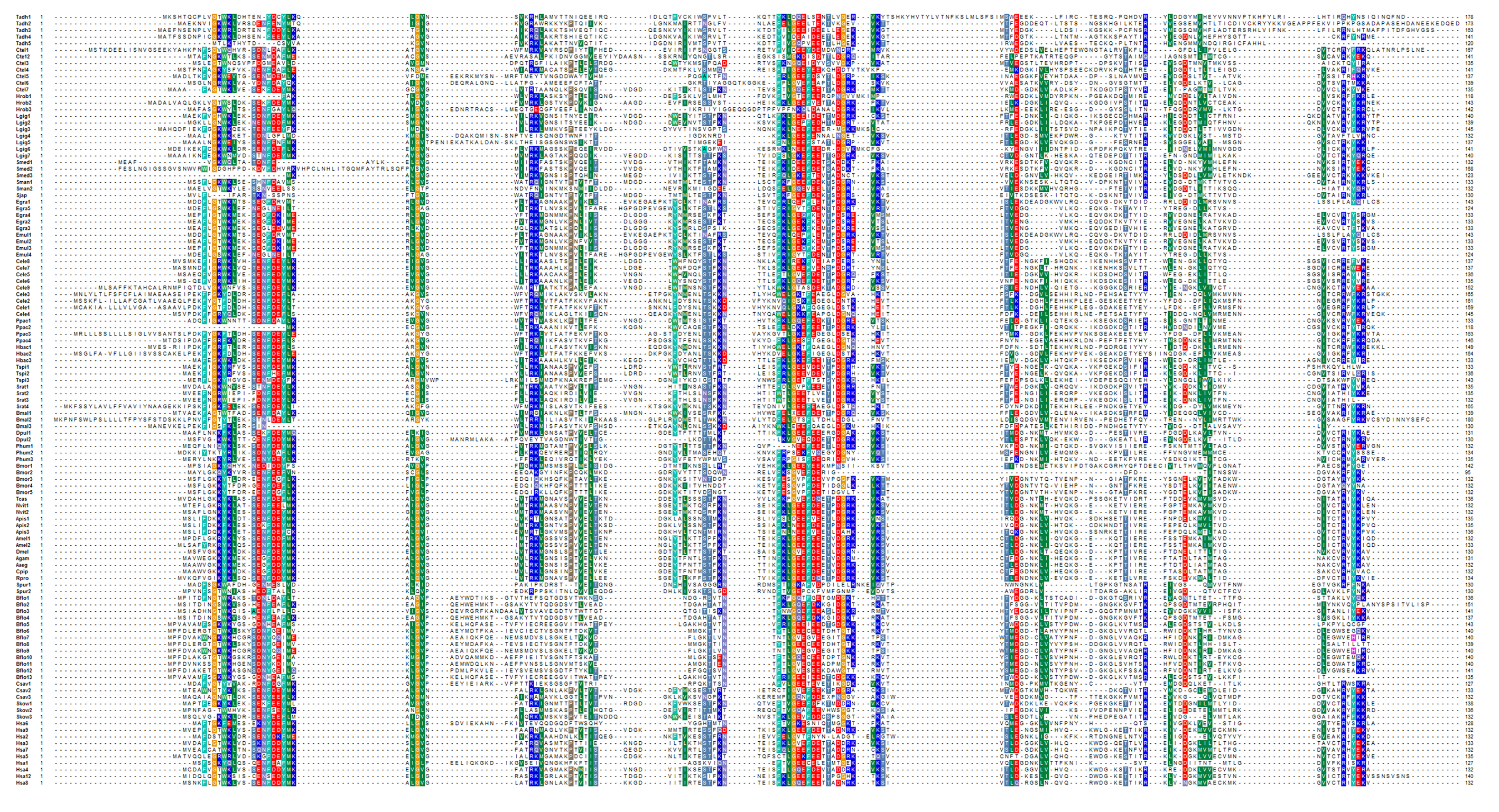

Supplement: Figure S1 — Alignment of FABP amino acid sequences. Tadh: Trichoplax adhaerens; Ctel: Capitella teleta; Hrob: Helobdella robusta; Lgig: Lottia gigantean; Smed: Schmidtea mediterranea; Sman: Schistosoma mansoni; Sjap: Schistosoma japonicum; Egra: Echinococcus granulosus; Emul: Echinococcus multilocularis; Cele: Caenorhabditis elegans; Ppac: Pristionchus pacificus; Hbac: Heterorhabditis bacteriophora; Tspi: Trichinella spiralis; Srat: Strongyloides ratti; Bmal: Brugia malayi; Dpul: Daphnia pulex; Phum: Pediculus humanus corporis; Bmor: Bombyx mori; Tcas: Tribolium castaneum; Nvit: Nasonia vitripennis; Apis: Acyrthosiphon pisum; Amel: Apis mellifera; Dmel: Drosophila melanogaster; Agam: Anopheles gambiae; Aaeg: Aedes aegypti; Cpip: Culex pipiens quinquefasciatus; Rpro: Rhodnius prolixus; Spur: Strongylocentrotus purpuratus; Bflo: Branchiostoma floridae; Csav: Ciona savignyi; Skow: Saccoglossus kowalevskii; Has: Homo sapiens Note: to make it simpler, ‘FABP’ was omitted in every branch name. For example: Tadh1 refers to Tadh_FABP1, Tadh2 to Tadh_FABP2 and so forth. (TIF) [file pone.0077636.s001.tif]

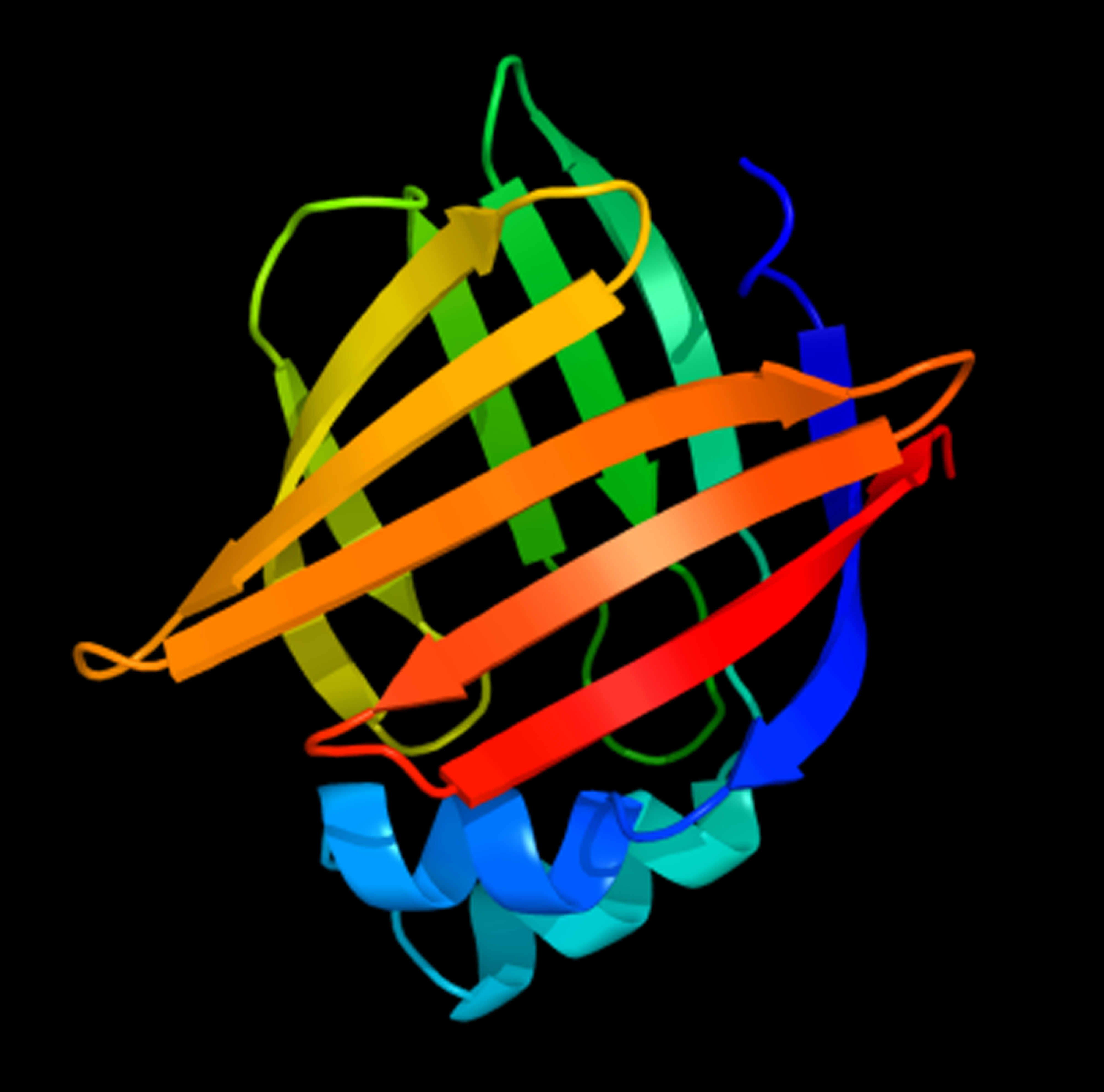

Supplement: Figure S2 — Tertiary structure of Emul_FABP3 predicted using Phyre. (TIF) [file pone.0077636.s002.tif]

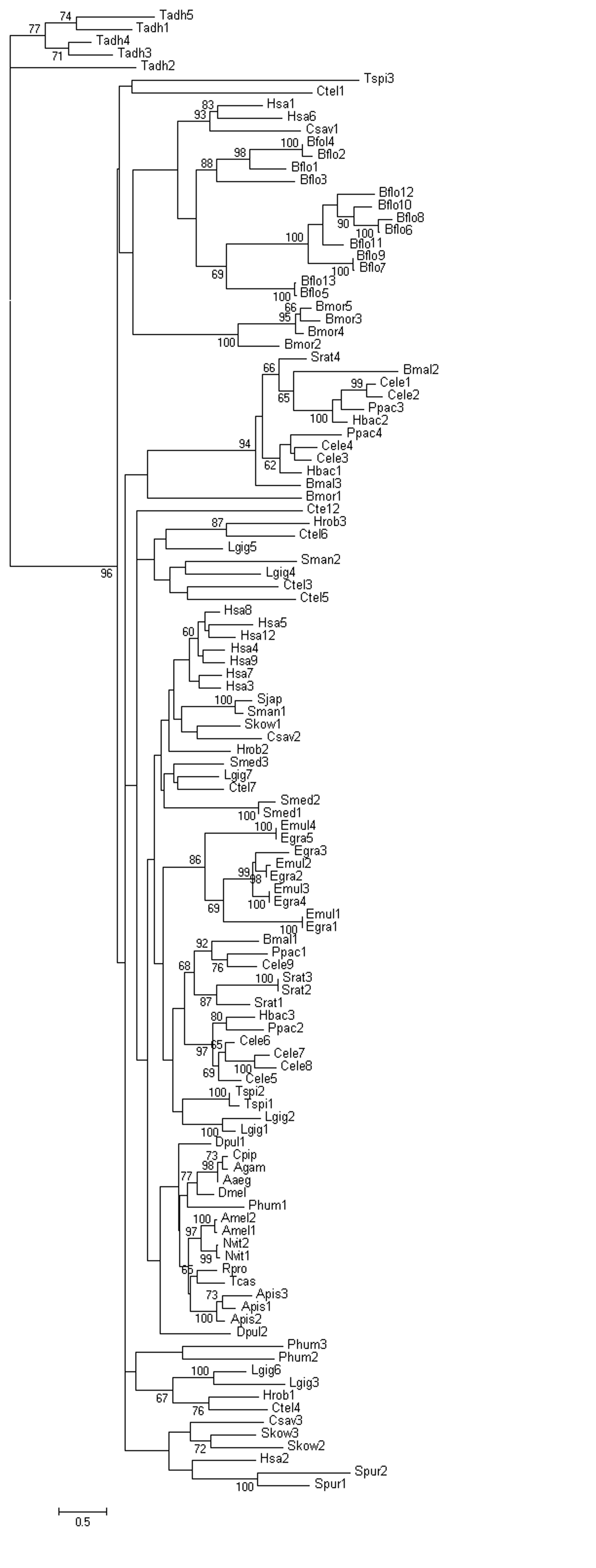

Supplement: Figure S3 — A ML tree of FABPs. Bootstrap values more than 60 were shown at nodes. Tadh: Trichoplax adhaerens; Ctel: Capitella teleta; Hrob: Helobdella robusta; Lgig: Lottia gigantean; Smed: Schmidtea mediterranea; Sman: Schistosoma mansoni; Sjap: Schistosoma japonicum; Egra: Echinococcus granulosus; Emul: Echinococcus multilocularis; Cele: Caenorhabditis elegans; Ppac: Pristionchus pacificus; Hbac: Heterorhabditis bacteriophora; Tspi: Trichinella spiralis; Srat: Strongyloides ratti; Bmal: Brugia malayi; Dpul: Daphnia pulex; Phum: Pediculus humanus corporis; Bmor: Bombyx mori; Tcas: Tribolium castaneum; Nvit: Nasonia vitripennis; Apis: Acyrthosiphon pisum; Amel: Apis mellifera; Dmel: Drosophila melanogaster; Agam: Anopheles gambiae; Aaeg: Aedes aegypti; Cpip: Culex pipiens quinquefasciatus; Rpro: Rhodnius prolixus; Spur: Strongylocentrotus purpuratus; Bflo: Branchiostoma floridae; Csav: Ciona savignyi; Skow: Saccoglossus kowalevskii; Has: Homo sapiens Note: to make it simpler, ‘FABP’ was omitted in every branch name. For example: Tadh1 refers to Tadh_FABP1, Tadh2 to Tadh_FABP2 and so forth. (TIF) [file pone.0077636.s003.tif]

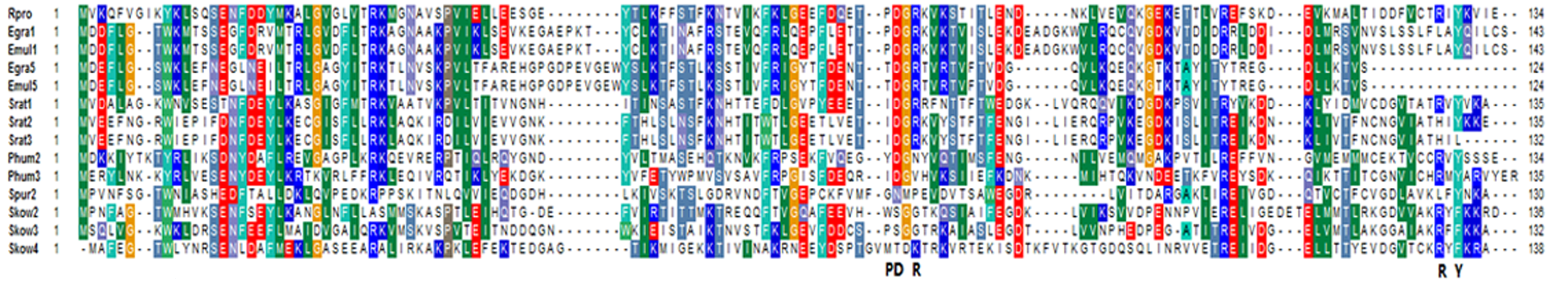

Supplement: Figure S4 — Fatty acid binding-related residues in intronless FABP genes of invertebrates. Invertebrate intronless FABP amino acid sequences were aligned using Clustal W. The amino acids identical to the consensus are shown as dots and alignment gaps are indicated with dashes (-). Numbers above the alignment represent positions of amino acids. The key amino acids responsible for interactions with lipid ligands are directly indicated beneath the alignment. Rpro, Rhodnius prolixus; Egra, Echinococcus granulosus; Emul, E. multilocularis; Srat, Strongyloides ratti; Phum, Pediculus humanus corporis; Spur, Strongylocentrotus purpuratus; Skow, Saccoglossus kowalevskii. Note: to make it simpler, ‘FABP’ was omitted in every branch name. For example: Tadh1 refers to Tadh_FABP1, Tadh2 to Tadh_FABP2 and so forth. (TIF) [file pone.0077636.s004.tif]
